# Optimization of a small molecule inhibitor of secondary nucleation in α-synuclein aggregation

**DOI:** 10.3389/fmolb.2023.1155753

**Published:** 2023-08-28

**Authors:** Roxine Staats, Z. Faidon Brotzakis, Sean Chia, Robert I. Horne, Michele Vendruscolo

**Affiliations:** Centre for Misfolding Diseases, Yusuf Hamied Department of Chemistry, University of Cambridge, Cambridge, United Kingdom

**Keywords:** Parkinson’s disease, drug discovery, secondary nucleation, kinetic theory, docking

## Abstract

Parkinson’s disease is characterised by the deposition in the brain of amyloid aggregates of α-synuclein. The surfaces of these amyloid aggregates can catalyse the formation of new aggregates, giving rise to a positive feedback mechanism responsible for the rapid proliferation of α-synuclein deposits. We report a procedure to enhance the potency of a small molecule to inhibit the aggregate proliferation process using a combination of *in silico* and *in vitro* methods. The optimized small molecule shows potency already at a compound:protein stoichiometry of 1:20. These results illustrate a strategy to accelerate the optimisation of small molecules against α-synuclein aggregation by targeting secondary nucleation.

## Introduction

Parkinson’s disease is the most prevalent neurodegenerative movement disorder (Poewe et al., 2017; Dorsey et al., 2018; Balestrino and Schapira, 2020; Aarsland et al., 2021). Although its pathogenesis involves a wide range of pathways and mechanisms, including mitochondrial dysfunction, oxidative stress, calcium dyshomeostasis, impairment of axonal transport and neuroinflammation, a major histopathological hallmark of the disease is the presence of α-synuclein aggregates known as Lewy bodies (Spillantini et al., 1997; Spillantini et al., 1998). These aggregates form through a complex kinetic process, which involves intertwined microscopic steps (Knowles et al., 2009; Buell et al., 2014). Small oligomeric assemblies form initially from monomeric precursors through primary nucleation, in a process often catalysed by lipid membranes (Galvagnion et al., 2015; Flagmeier et al., 2016; Galvagnion et al., 2016). The oligomers, which are initially disordered, can convert into ordered forms (Cremades et al., 2012), which can grow into long fibrils through monomer-dependent elongation (Knowles et al., 2009; Buell et al., 2014; Galvagnion et al., 2015; Flagmeier et al., 2016; Galvagnion et al., 2016). The presence of these fibrils can then catalyse the formation of new oligomeric assemblies, in an autocatalytic process responsible for the rapid proliferation of α-synuclein deposits (Knowles et al., 2009; Buell et al., 2014; Galvagnion et al., 2015; Flagmeier et al., 2016; Galvagnion et al., 2016). This process is known as secondary nucleation because it depends on the presence of already formed amyloid fibrils, and it is typically much faster than primary nucleation.

Since the aggregation of α-synuclein is cytotoxic, in particular through the formation of the oligomeric intermediates (Winner et al., 2011; Fusco et al., 2017; Cascella et al., 2021), many drug discovery programs have targeted this process (Wagner et al., 2013; Price et al., 2018; Pujols et al., 2018; McFarthing et al., 2020; Staats et al., 2020; Lang et al., 2022; Pagano et al., 2022; Chia et al., 2023; Horne et al., 2023). Here we present a technique to identify and rationally optimise a small molecule that binds α-synuclein fibrils by utilising a hybrid *in silico* and *in vitro* approach. This pipeline is initiated by identifying candidate molecules in a virtual screen, wherein small molecules are ranked according to their binding energy score with the α-synuclein fibrillar surface (Chia et al., 2023). In an affinity optimisation step, these top ranked compounds are derivatised, and subsequently tested in a series of α-synuclein aggregation assays in order to characterise their mechanism of action.

Quite generally, compounds that bind the surface of the fibrils at the catalytic sites for secondary nucleation could act as inhibitors of fibril proliferation (Michaels et al., 2020a; Michaels et al., 2022). This mechanism of action has significant potential therapeutic implications, as the secondary nucleation process underpins the formation of oligomeric species (Cohen et al., 2013; Michaels et al., 2020b; Linse et al., 2020; Michaels et al., 2022), some of which can be neurotoxic (Haass and Selkoe, 2007; Winner et al., 2011; Benilova et al., 2012; Fusco et al., 2017; Cascella et al., 2021). Thus, we measure the propensity of the compounds to inhibit this process in thioflavin T (ThT) aggregation assays, which serve as a basis to model the flux towards oligomeric species formation (Staats et al., 2020).

## Results

### Characterisation of the parent compound 69.0

Small molecules from the ZINC library were screened *in silico* for their ability to dock into a pocket on the α-synuclein fibril surface using AutoDock Vina and FRED (Chia et al., 2023). This pocket comprises residues 43–58 of α-synuclein and, in the context of the fibrillar structure (Li et al., 2018), this section of the monomeric protein represents the constituent residues at an interface between two protofibrils. This pocket has previously been identified as a putative small molecule binding site in similar docking studies (Hsieh et al., 2018). One such compound that exhibited favourable docking parameters *in silico* was the parent compound 69.0 (Chia et al., 2023) (Supplementary Figure S1). We selected this compound here because of its numerous purchasable derivatives. To obtain a more detailed characterization of the bound state, we equilibrated the bound state of compound 69.0 within the pocket in the α-synuclein fibril using a short molecular dynamics simulation (see Methods). The small molecule occupies the binding pocket comprising residues K43, K45, H50, E57, K58 and forms hydrogen bonds between the carbonyl groups of the phenylpyrazolidinedione moiety with K45 and K58. The phenyl group is sandwiched between the lysine residues K45 and K58, as well as glutamic acid E57 and histidine H50. Importantly, this binding pocket is conserved in amyloid fibrils from Parkinson’s brains (Yang et al., 2022).

Following its favourable result in a docking simulation with the α-synuclein fibril structure, compound 69.0 was tested in an α-synuclein secondary nucleation reaction *in vitro* to ascertain whether its predicted docking affinity to the fibril surface corresponded with an ability to inhibit surface-catalysed fibril amplification (Chia et al., 2023) (Supplementary Figure S2). The α-synuclein secondary nucleation assay is conducted under mildly acidic conditions, leading to the formation of oligomeric species and fibril amplification (Buell et al., 2014). The assay occurs under quiescent conditions, allowing for quantitative kinetic analyses to be performed on the resulting readout. This assay was chosen as it is mechanistically best suited to evaluate the potential inhibitory potency of a fibril-binding compound, as the inhibition of secondary nucleation may function as a readout of the ability of a given compound to bind the autocatalytic nucleation sites along the α-synuclein fibril surface. To this end, the compound was incubated with 20 µM α-synuclein monomer and 50 nM preformed fibrils at 0.25, 0.5 and 1 M equivalents relative to monomeric protein and the ThT trace followed for approximately 145 h (Supplementary Figure S2A). The normalised data were then fitted and the fibril amplification rate for each condition was extracted using Eqs 2, 6 (see Methods and Supplementary Figure S2B). The compound was able to elicit a ∼82% and ∼93% reduction in the fibril amplification rate of α-synuclein at 0.25 and 0.5 M equivalents, respectively, while 1 M equivalent of compound with respect to monomeric α-synuclein prevented aggregation entirely.

### Derivatisation by catalogue of the parent compound 69.0

**FIGURE 1:**
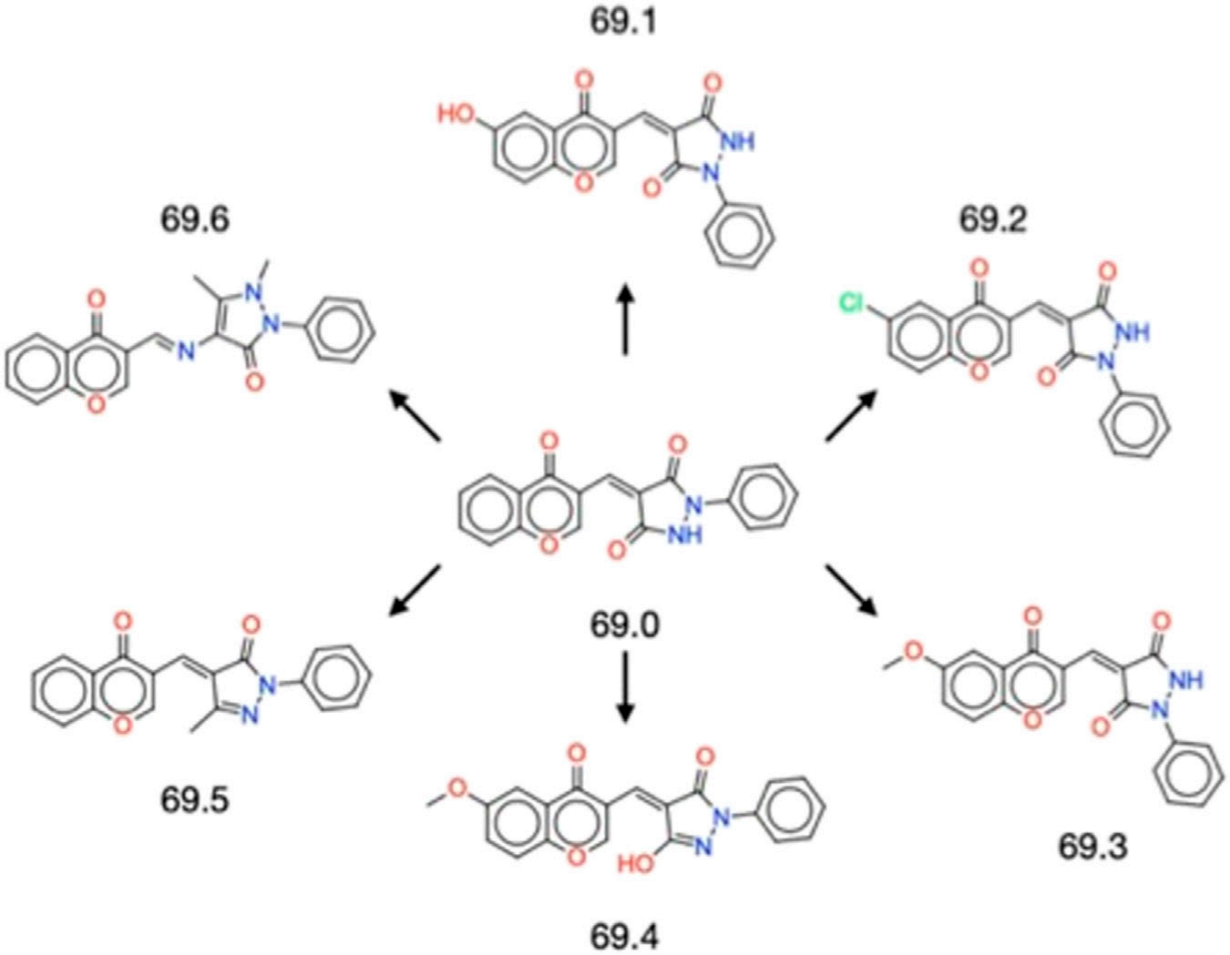
Structures of the 6 derivatives of the parent compound 69.0 studied in this work. Molecules 69.1–69.3 alter only the chromenone portion of the central scaffold, exploring hydroxylation, chlorination and methoxylation, respectively. Molecule 69.4 is a close derivative of molecule 69.3, but it alters the phenylpyrazolidinedione moiety of the parent to become a hydroxyphenylpyrazolone. Molecules 69.5 and 69.6 maintain the unaltered chromenone region of the parent scaffold, but constitute a methylated phenylpyrazolone and a dimethylated phenylpyrazolone, respectively, where molecule 69.6 contains a methylethanimine group linking the pyrazolone to the chromenone region.

The successful validation of the parent compound 69.0 as an inhibitor of α-synuclein secondary nucleation prompted us to derivatise it to identify chemical alterations that may confer enhanced potency against α-synuclein aggregation (Figure 1). The parent compound contains a central scaffold comprised of a chromenone and a phenylpyrazolidinedione moiety. A similarity search resulted in 6 derivatives (69.1–69.6). Molecules 69.1, 69.2 and 69.3 alter only the chromenone portion of the central scaffold, exploring hydroxylation, chlorination and methoxylation, respectively. Molecule 69.4 is a close derivative of molecule 69.3, but it alters the phenylpyrazolidinedione moiety of the parent to become a hydroxyphenylpyrazolone. Molecules 69.5 and 69.6 maintain the unaltered chromenone region of the parent scaffold, but constitute a methylated phenylpyrazolone and a dimethylated phenylpyrazolone, respectively, where molecule 69.6 contains a methylethanimine group linking the pyrazolone to the chromenone region.

### Effects of the 6 derivatives on α-synuclein secondary nucleation

**FIGURE 2:**
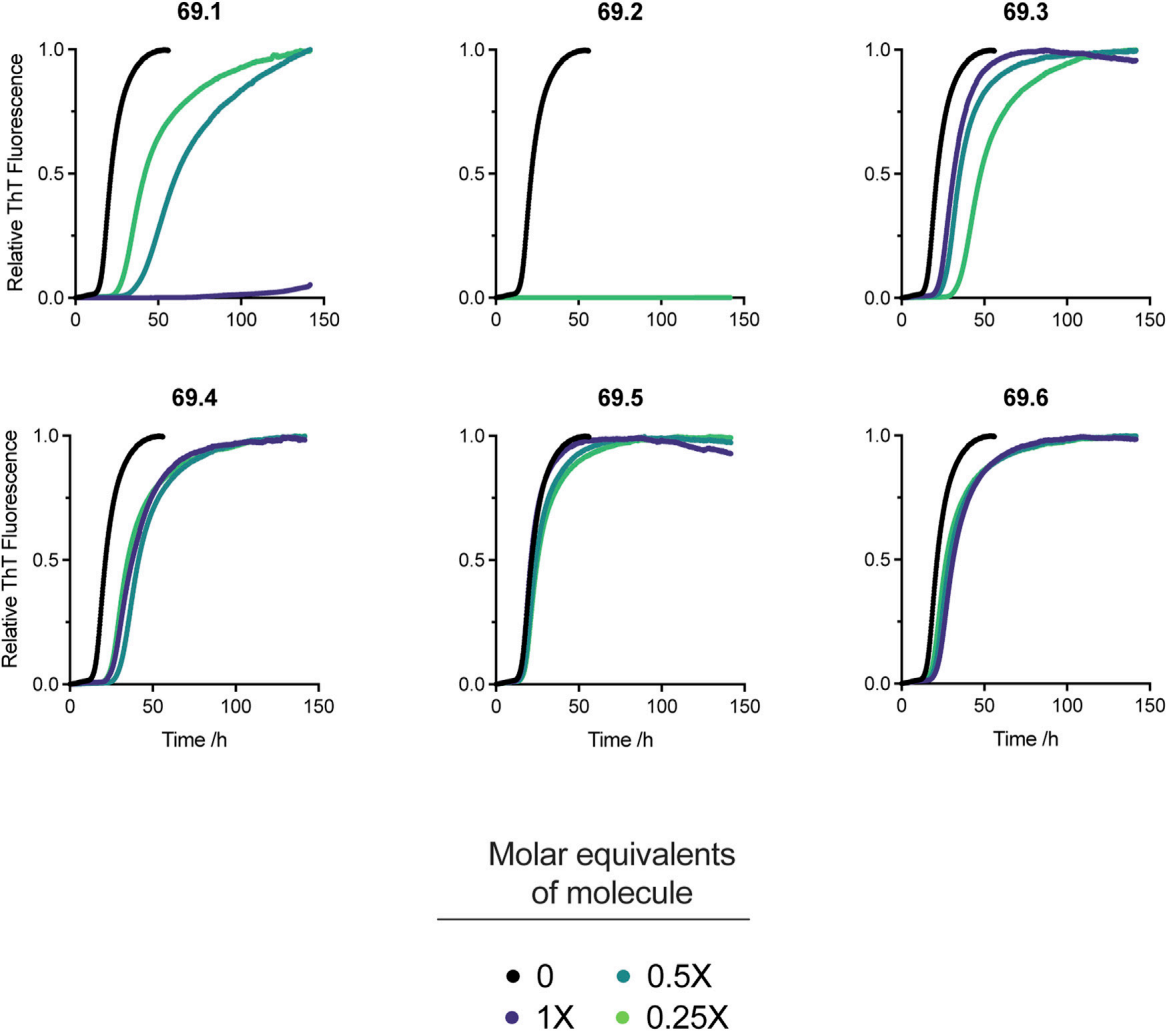
Effects on α-synuclein secondary nucleation of the 6 derivatives of the parent compound 69.0. Change in ThT fluorescence when 20 μM α-synuclein monomer was incubated in the absence and presence of derivatives 69.1–69.6 at 37°C under quiescent conditions with 0.25% seed fibrils in sodium phosphate buffer (20 mM, pH 4.8, 1% DMSO) with increasing compound concentrations as indicated. Traces indicate the mean and error of three technical repeats. Compound 69.2 completely inhibited α-synuclein at all the concentrations tested.

The 6 derivatives of the parent compound 69.0 were assayed in the α-synuclein secondary nucleation assay to assess whether any had enhanced potency against this process. Of these candidate molecules, compound 69.2 was observed to have markedly increased efficacy in the secondary nucleation assay, as opposed to its parent structure, or any other compound within the derivatisation cluster (Figure 2).

### Docking of the derivative compound 69.2

**FIGURE 3:**
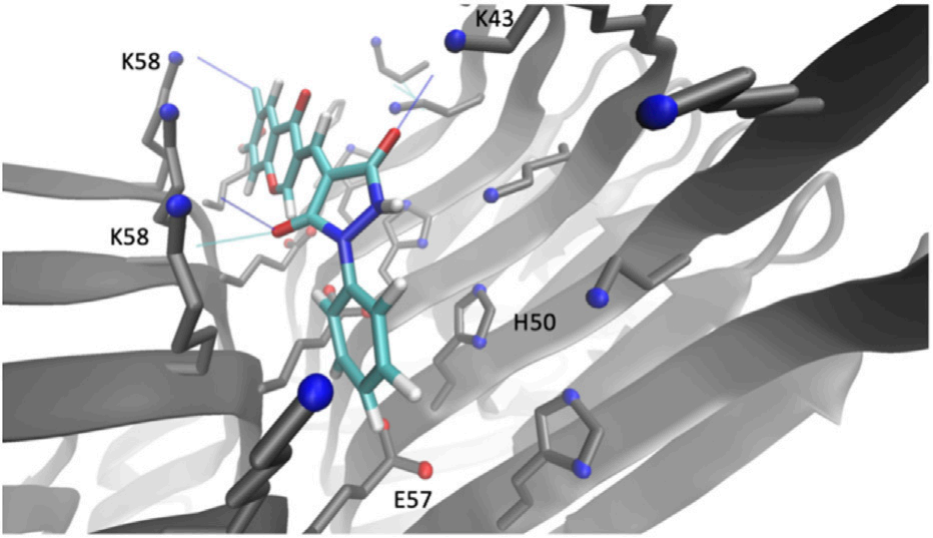
Binding pocket of compound 69.2 on the surface of an α-synuclein fibril structure. Illustration of the best docking pose and the respective interactions of the derivative compound 69.2 within a pocket on the surface of an α-synuclein fibril structure (PDB 6CU7) comprising residues K43, K45, H50, E57, K58.

Compound 69.2 was virtually docked to the α-synuclein fibril structure to investigate how the modifications made to the parent scaffold modified the interactions within the identified pocket (Figure 3). The compound was equilibrated in a short molecular dynamics simulation (see Methods) and found to form similar interactions with the fibril structure as compared to its parent compound (Supplementary Figure S1), occupying the binding pocket comprising residues K43, K45, H50, E57, and K58. The compound was observed in the simulations to form hydrogen bonds between the carbonyl groups of the phenylpyrazolidinedione moiety with residues K43 and K58, while its constituent phenyl group is sandwiched between the lysine residues K45 and K58, as well as the glutamic acid residue E57 and histidine residue H50. Overall, the compound appears to be buried within a larger hydrophobic surface area than in the case of the parent. Notably, the addition of the chlorine group introduced a new stabilising halogen bond interaction with K58, possibly explaining the higher affinity. The average binding energy of compound 69.2 over all poses was −5.7 ± 1.2 kcal/mol. This average binding energy can be compared with that of the parent compound 69.0, which was −3.6 ± 1.0 kcal/mol. The other derivatives of compound 69.0 exhibited an intermediate behaviour between these values.

### Kinetic characterisation of the derivative compound 69.2

We thus decided to conduct an extensive kinetic profiling of compound 69.2 to gain insight into the degree of substoichiometry, with respect to α-synuclein monomer, that would yield potency in the inhibition of α-synuclein aggregation, as well as to provide further evidence that the compound did indeed confer specificity for secondary nucleation inhibition by the intended mechanism of action of binding to catalytic sites along the fibril surface. Thus, in addition to an *in silico* and *in vitro* validation of the ability of the derivative compound to dock the α-synuclein fibril surface, we conducted α-synuclein lipid-induced aggregation assays with a 1,2-dimyristoyl-sn-glycero-3-phospho-L-serine (DMPS) model membrane system (Galvagnion et al., 2015; Flagmeier et al., 2016; Galvagnion et al., 2016), as well as a fibril elongation assay (Buell et al., 2014; Flagmeier et al., 2016), to ascertain the mechanistic specificity of the derivative compound and to gain additional kinetic insights.

#### Compound 69.2 confers potency against α-synuclein secondary nucleation

**FIGURE 4:**
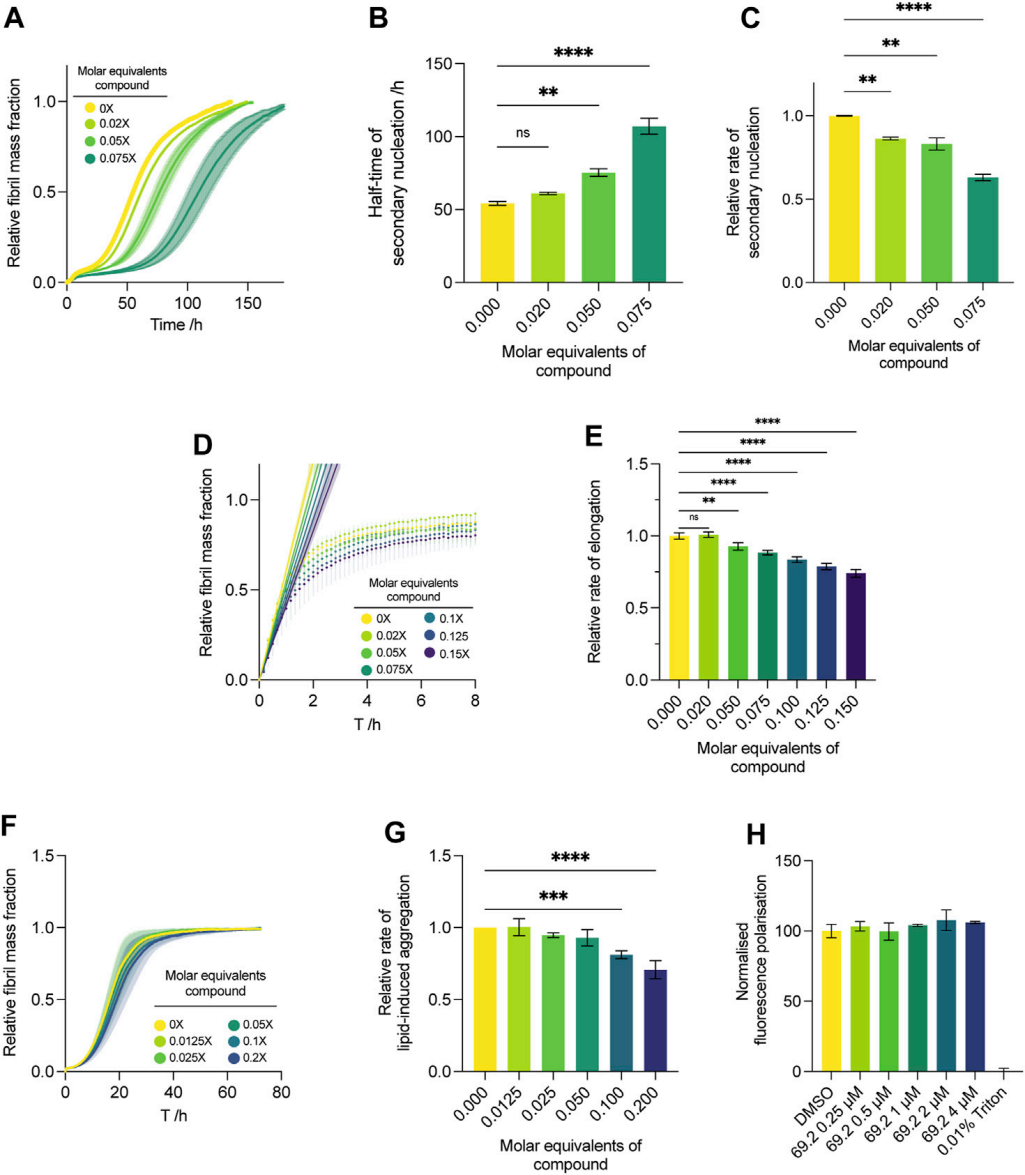
Effect of compound 69.2 on α-synuclein secondary nucleation. **(A)** Change in ThT fluorescence when 50 μM α-synuclein monomer was incubated at 37°C under quiescent conditions with 0.06% seed fibrils in MES buffer (10 mm, 1 mm EDTA, pH 5.5, 1% DMSO) with increasing compound concentrations as indicated. The shaded bands represent the standard error of the mean between three experiments each containing three technical replicates. **(B)** Half-time of the α-synuclein secondary nucleation reaction. **(C)** Effective rate of α-synuclein fibril amplification, normalised relative to the DMSO control. Error bars represent the standard error of the mean of three experimental replicates each containing three technical replicates. Statistical analyses represent ordinary one-way ANOVA results where **, *** and **** indicate a multiplicity-adjusted *p*-value of ≤0.01, 0.001 and 0.0001, respectively. **(D)** Effect of compound 69.2 on α-synuclein fibril elongation. Normalised change in ThT fluorescence when 50 μM monomeric α-synuclein was incubated with 7% preformed seed fibrils relative to monomer at pH 5.5 and 37°C in the absence (yellow trace) and presence of the derivative compound 69.2 at the concentrations indicated. Solid straight lines indicate linear fits to the initial phases of the reaction to obtain the effective rate of elongation. **(E)** Effective rate of α-synuclein fibril elongation, normalised relative to the DMSO control. Error bars represent the standard error of the mean of three experimental replicates each containing three technical replicates. Statistical analyses represent ordinary one-way ANOVA results where ** and **** indicate a multiplicity-adjusted *p*-value of ≤0.01 and 0.0001, respectively. **(F)** Effect of compound 69.2 on α-synuclein lipid-induced aggregation. Change in ThT fluorescence when 20 μM α-synuclein was incubated with 100 μM DMPS vesicles in 20 mM sodium phosphate buffer (pH 6.5) at 30°C under quiescent conditions with increasing concentrations of compound 69.2. **(G)** Relative rate of lipid-induced aggregation extracted for each concentration of compound by fitting a one-step nucleation model (Eq. 12) to the early time points of the time course. Error bars represent the standard error between three experiments each containing three technical repeats. Statistical analyses represent ordinary one-way ANOVA results where *** and **** indicate a multiplicity-adjusted *p*-value of ≤0.001 and 0.0001, respectively. **(H)** Diphenylhexatriene (DPH) fluorescence polarisation measurements to determine DMPS membrane fluidity changes upon addition of compound 69.2. No changes in membrane fluidity were observed over a concentration range of up to 4 μM inhibitor (with 100 µM lipid vesicle). 100% disruption is represented by the addition of 0.01% Triton.

We conducted the secondary nucleation assay with a lower concentration gradient of the compound of interest (Figure 4). As our initial screen (Figure 2), which spanned 0.25–1 M equivalents of the compound with respect to monomeric α-synuclein, yielded complete inhibition of aggregation at all concentrations assayed and resulted in a flat line with respect to the ThT readout, no quantitative kinetic parameters could be extracted. Thus, we repeated the assay and lowered the compound concentration range approximately tenfold, yielding more insight into the dose-dependent effects of the compound on the system. The results indicate that the compound maintains its potency at very low stoichiometry, yielding a delay in the increase of ThT fluorescence at all concentrations (Figure 4A). Compound 69.2 was able to delay the half-time of the aggregation by 6.9 h, or ∼12.5% relative to the control sample, although this was not significant. However, the compound was able to delay the half-time of the aggregation significantly by ∼41% and ∼100% at 0.05 and 0.075 M equivalents with respect to monomeric α-synuclein, respectively (Figure 4B). In addition, the compound was able to elicit a significant drop in the rate of α-synuclein fibril amplification, reducing the rate of secondary nucleation by ∼14%, 17%, and 37% at 0.02 and 0.05 and 0.075 M equivalents, respectively (Figure 4C).

#### Compound 69.2 only mildly inhibits α-synuclein fibril elongation

Under conditions where the initial preformed fibril concentration is elevated to about 10% of the initial monomer concentration, the aggregation is skewed towards an elongation-dominated process (Buell et al., 2014). This reveals the propensity of a given compound to inhibit the fibril elongation process wherein monomeric protein is recruited on to fibril ends to form mature aggregate species. Although the compound was able to inhibit the elongation process slightly, the effect was not as pronounced as its ability to delay secondary nucleation (Figure 4). After following the increase in ThT fluorescence under elongation-dominated conditions for approximately 8 h (Figure 4D), quantitative kinetic analysis was performed as per Eq. 1, and the compound was found to elicit a statistically significant drop of ∼26% at 0.15 M equivalents relative to monomer, representing the maximum concentration assayed (Figure 4E).

#### Compound 69.2 only mildly inhibits α-synuclein lipid-induced aggregation

While the physiological functions of α-synuclein involve interactions with cellular membranes, it is possible that aberrant interactions with such surfaces and sufficient local concentration excess of the protein may induce aggregation within physiological systems (Galvagnion et al., 2015; Fusco et al., 2016; Galvagnion et al., 2016; Fusco et al., 2017). By introducing monomeric α-synuclein in the presence of DMPS lipid vesicles, we model this process. Under the conditions required for this assay, secondary nucleation via catalysis on fibrillar surfaces that arise during the bulk aggregation process are thought to play only a minor role during the initial, exponentially increasing phase of the aggregation, allowing the rate of lipid-induced aggregation to be approximated using a one-step nucleation model (Eq. 12) (Galvagnion et al., 2015). In the context of this study, this assay represents a useful tool for substantiating the presumed mechanism of action of the compound of interest. A lack of efficacy in this assay indicates that compound 69.2 inhibits secondary nucleation via direct interaction with fibril surfaces, rather than with monomeric α-synuclein. Indeed, compound 69.2 was not able to inhibit the lipid-induced aggregation assay to the same degree as the seeded secondary nucleation assay (Figure 4F), eliciting a significant reduction in the rate of lipid-induced aggregation only at the highest two doses assayed. The compound reduced the rate of lipid-induced aggregation by ∼20% and ∼29% at 0.1 and 0.2 M equivalents relative to α-synuclein monomer, respectively (Figure 4G). Incubation of the compound with lipid vesicles did not yield noticeable changes in membrane fluidity (Figure 4H) when measured with a diphenylhexatriene (DPH) fluorescence polarisation assay. This result suggests that the aggregation inhibition observed at high concentrations of compound 69.2 is likely to be mediated by the interaction of the compound with α-synuclein, rather than by the compound disrupting lipid vesicles.

#### Compound 69.2 reduces the oligomeric flux

During α-synuclein secondary nucleation, where new fibrils are formed by means of autocatalytic nucleation on existing fibril surfaces, α-synuclein forms a transient oligomeric state before maturing into the stable fibrillar structure (Michaels et al., 2020b). This process highlights a key point of interest in the therapeutic intervention to inhibit secondary nucleation, as these oligomeric species confer marked cellular toxicity (Haass and Selkoe, 2007; Winner et al., 2011; Benilova et al., 2012; Fusco et al., 2017; Cascella et al., 2021). Delaying their onset or decreasing the rate at which they are able to form represent potential therapeutic interventions that may ameliorate aggregate toxicity in the disease state (Cohen et al., 2013; Michaels et al., 2020b; Linse et al., 2020; Michaels et al., 2022). This rate of formation, which corresponds to a flux of the system towards the formation of oligomeric species, is mediated both by the effective rate of secondary nucleation and the effective rate of elongation within the system (Staats et al., 2020). This is owing to the process of secondary nucleation acting as a source of new oligomeric species, while the process of fibril elongation converts these species into mature fibrillar material, thereby acting as a sink (Michaels et al., 2020a; Michaels et al., 2022). Thus, by considering the interplay between the effective rates of fibrillar amplification and elongation, we may delineate an approximation of the flux of the system towards oligomeric species formation.

**FIGURE 5:**
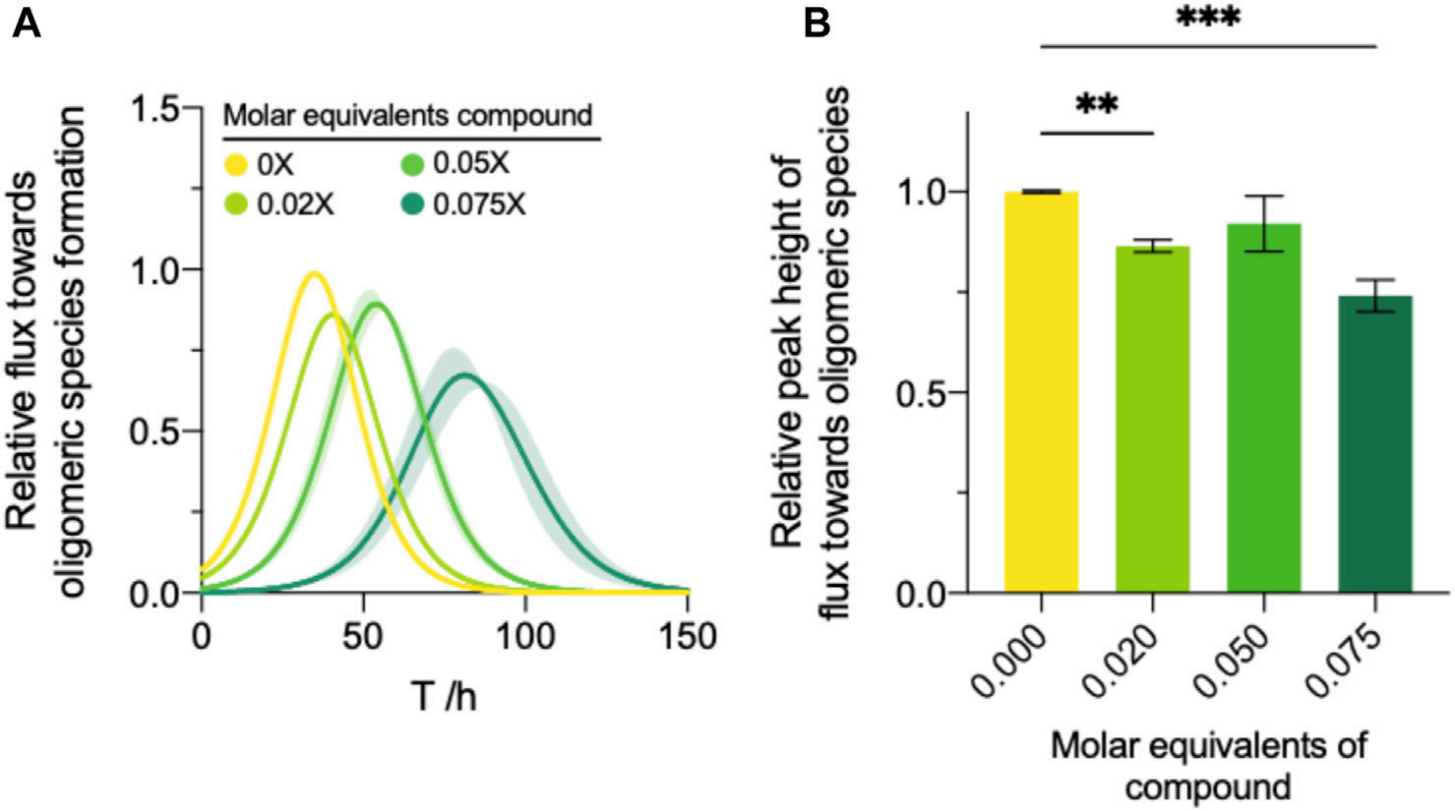
Modulation of the oligomer dynamics by compound 69.2. **(A)** The reactive flux towards oligomeric species over time, φ, relative to the DMSO control for each compound. **(B)** Normalised area under the curve of the reactive flux towards α-synuclein oligomer formation relative to the DMSO control. Error bars represent the standard error of the mean of three experimental replicates each containing three technical replicates. Statistical analyses represent ordinary one-way ANOVA results where **, ***, and **** indicate a multiplicity-adjusted *p*-value of ≤0.01, 0.001 and 0.0001, respectively.

To this end, we applied the extracted effective rate quantifications of both secondary nucleation and elongation to Eq. 7 to yield an approximation of the oligomeric flux over time (Staats et al., 2020). Compound 69.2 was able to delay the formation of oligomeric species significantly (Figure 5A). The compound was able to reduce the peak height of *ϕ* (Eq. 11) by ∼14% and ∼26% at 0.02 and 0.075 M equivalents relative to monomer, respectively (Figure 5B).

#### Absorbance determination and label-free assessment of secondary nucleation inhibition by compound 69.2

Incubating α-synuclein fibrils in the presence of ThT and compound 69.2 showed that, at the maximum concentration assessed for secondary nucleation inhibition, compound 69.2 quenches the fluorescence of ThT by approximately 23% (Supplementary Figure S3). This perturbation in fluorescence may be accounted for by normalising the fluorescence readouts, as has been done in this work. Additionally, we adopted an orthogonal, label free methodology to validate the observed effects on α-synuclein aggregation by the compound. To assess the validity of the observed half-time delay induced by the compound in the ThT assay, we repeated the assay by nephelometry, which is label-free and detects the size of light-perturbing species, in this case aggregated α-synuclein, in solution (see Methods and Supplementary Figure S4). The results from this assay show that the effect of the compound is indeed observable under these conditions as well, which is consistent with the changes to secondary nucleation observed by ThT.

### Determination of the binding affinity of compound 69.2 by mass spectrometry

**FIGURE 6:**
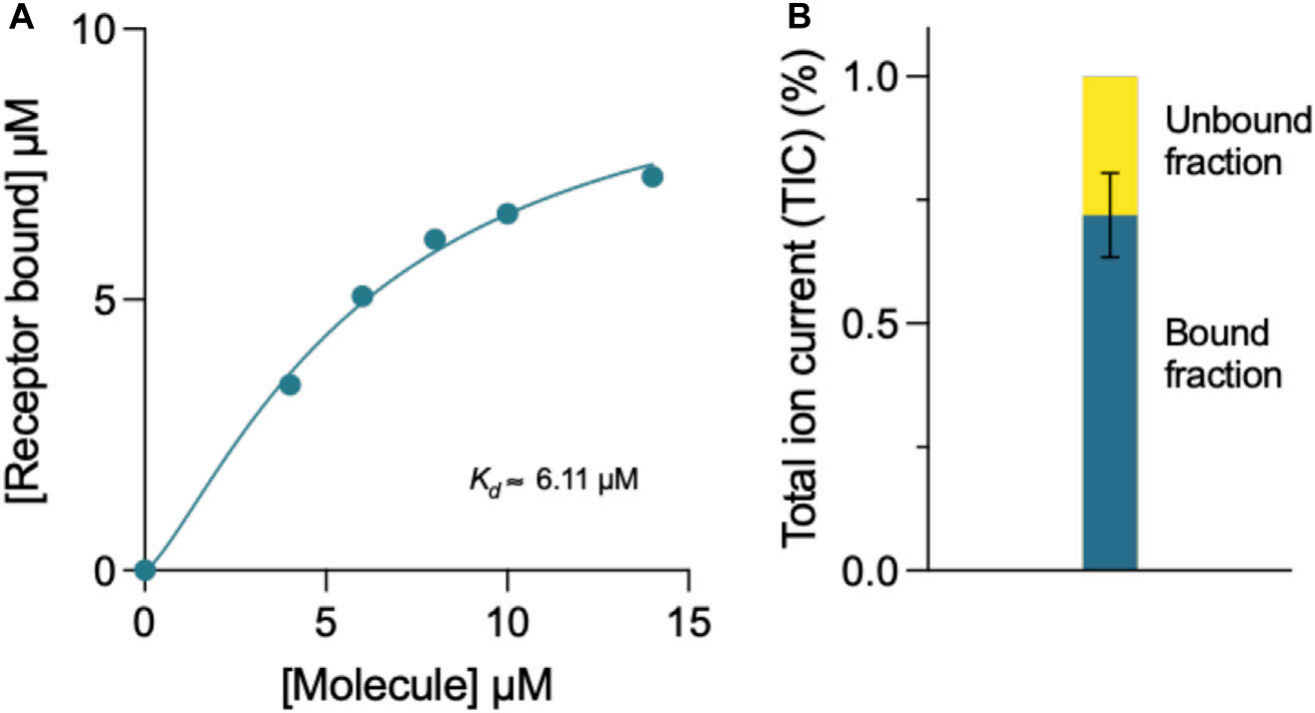
Mass spectrometry determination of the binding affinity of compound 69.2 for α-synuclein fibrils. **(A)** Concentration of α-synuclein fibrils bound in the presence of increasing concentrations of compound 69.2. From the fit of this dataset, we estimated the binding affinity of the compound for the fibrils to be 6 ± 1 μM. By repeating the experiments by varying the concentration of the fibrils, we found a binding affinity of 3 ± 1 μM. At each fibril concentration, the error was estimated form the fitting procedure. **(B)** Total ion current (TIC) of 10 μM compound 69.2 bound and unbound to 10 μM α-synuclein fibrils detected by mass spectrometry.

The binding affinity of compound 69.2 to the α-synuclein fibril surface was evaluated using mass spectrometry (Figure 6). The compound was incubated at varying concentrations with a constant concentration of α-synuclein preformed fibrils, after which samples were ultracentrifuged and the compound left in the supernatant assessed by liquid chromatography - mass spectrometry (LC-MS) (Figure 6A). Fitting a four-parameter dose-response curve (Eq. 13) to the data, where the maximum response was constrained to the concentration of receptor assayed (10 µM preformed α-synuclein fibrils), the fit resulted in a binding affinity of in the 3–6 µM range (Figure 6A). With this method, the incubation of the derivative compound with an equal concentration of α-synuclein fibrillar material revealed that approximately 72% of the compound was bound to fibrillar material under these conditions (Figure 6B).

### Conclusion

**FIGURE 7:**
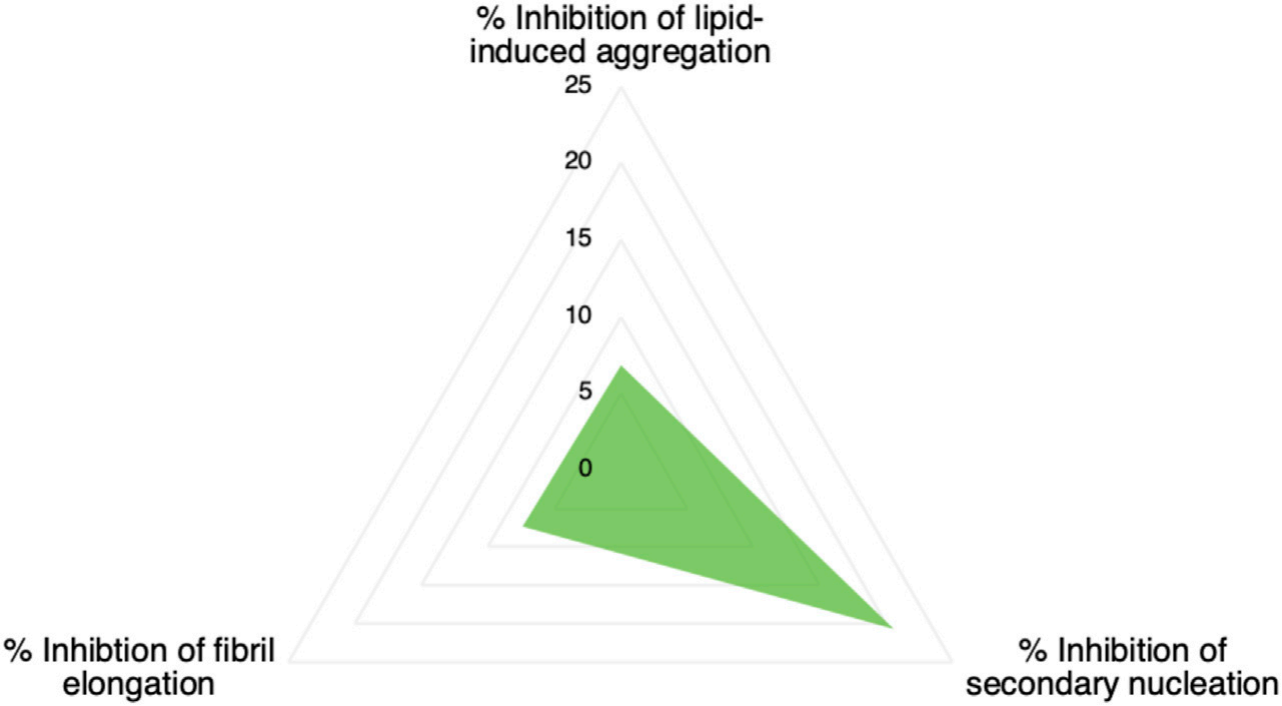
Inhibition of microscopic processes of α-synuclein aggregation by compound 69.2. The values refer to a 1:20 stoichiometry of compound:protein (0.05 M equivalents of the compound with respect to of α-synuclein monomer).

We have described a method of optimising an aggregation inhibitor of α-synuclein using a combination of structure-based and kinetic-based methods. We have first employed an *in silico* docking method to identify compounds to bind a pocket on the α-synuclein fibril surface that may act as catalytic site for secondary nucleation of α-synuclein aggregation. Then, we have identified one of these compounds with good ability to inhibit α-synuclein secondary nucleation *in vitro*. Finally, we have modified this compound by means of chlorination of its constituent chromenone region and showed that this optimization markedly improved its potency against α-synuclein secondary nucleation. The conjecture that this compound achieves this potency by binding to catalytic sites on the α-synuclein fibril surface is supported by the relative inactivity of the compound against α-synuclein fibril elongation and lipid-induced aggregation (Figure 7). Furthermore, the compound was observed to bind preformed α-synuclein fibrils by mass spectrometry.

Taken together, the results that we have reported indicate that molecule 69.2 acts as a fibril-binding inhibitor of α-synuclein secondary nucleation and oligomer formation. In perspective, the approach that we have described positions amyloid fibrils within the remit of structure-based drug discovery methods.

## Methods

### In silico docking to the α-synuclein fibril surface

To identify the docking pose, the respective binding energy and the molecular interactions of the parent compound 69.0 and of the derivative 69.2, molecular docking was performed using Autodock Vina (Trott and Olson, 2010). The binding site around H50 and was determined by using the Fpocket (Le Guilloux et al., 2009) software against α-synuclein PDB structure (PDB ID: 6CU7). A cubic box of 1.2 nm × 1.2 nm x 0.9 nm, centered at the CE1 atom of H50 was used. This was partly motivated by the rationale that the autocatalytic secondary nucleation of α-synuclein is favoured below pH 6, prompting the exploration of binding pockets rich in amino acids possessing a pKa of around 6. The binding energy predictions and standard deviations reported here are based on a sample of 20 generated poses. In order to refine the docked pose interactions of compounds, a short explicit water molecular dynamics simulations starting from the best pose were performed using GROMACS (Danne et al., 2017) with the Amber99SB-ILDN (Lindorff‐Larsen et al., 2010), TIP3P (Jorgensen et al., 1983) and GAFF (Wang et al., 2004) for the protein, water and ligand force fields, respectively. The system was subsequently energy minimized, equilibrated in an NPT and subsequent NVT molecular dynamics equilibration. To constrain bond lengths, the LINCS algorithm was employed (Hess, 2008). The Lennard-Jones interactions were treated with a 1 nm cut-off, while the electrostatic interactions are treated with the Particle Mesh Ewald method using a Fourier spacing of 1.2 nm and a 1 nm cut-off for the short-range electrostatic interactions. Pair lists are updated every 10 fs, using a cut-off of 1 nm and a time step of 2 fs Integration of Newton’s equations of motion is performed using the leap-frog algorithm (Frenkel and Smit, 2001), the velocity-rescaling thermostat (Bussi et al., 2007) with a coupling time constant of 0.2 ps, and the Parrinello-Rahman barostat (Parrinello and Rahman, 1981) for equilibration utilising a coupling time constant of 1.0 ps during NPT simulations. In the NPT equilibration, positions of Cα atoms were restrained with a constant force of 200 kJ/mol nm^2^, temperature was set to 310 K, the pressure to 1 atm and the simulation duration to 500 ps In the NVT equilibration we lifted the position restraints, simulated for 2 ns

### Reagents

#### Recombinant α-synuclein expression

Recombinant α-synuclein was expressed and purified as described previously. *Escherichia coli* BL21 Gold (DE3) cells were transformed with a human α-synuclein-encoding pT7-7 plasmid and grown in LB (2xγT) media in the presence of ampicillin (100 μg/mL). Cells were induced with 1 mM IPTG, cultured at 37 °C overnight and harvested by centrifugation in a Beckman Avanti J-20 centrifuge with a JLA-8.1000 rotor for 20 min at 4,000 rpm (Beckman Coulter, Fullerton, CA) and 4°C. The cell pellet was resuspended in 10 mM Tris–HCl (pH 7.7), 1 mM EDTA and lysed by sonication. The cell suspension was centrifuged for 20 min at 18,000 rpm at 4°C and the supernatant subsequently boiled by suspension in a water bath at 80°C–95°C for 20–25 min. The boiled supernatant was once again centrifuged for 20 min at 18,000 rpm at 4°C to pellet heat-denatured proteins. 10 mg/mL streptomycin sulphate was added to the supernatant to precipitate DNA and rolled for 15 min at 4°C on a benchtop rolling system. To pellet precipitated DNA, the mixture was centrifuged at for 20 min at 18,000 rpm at 4°C and the supernatant collected. To precipitate α-synuclein, ammonium sulphate was added to the supernatant to yield a final concentration of 361 mg/mL and the mixture was rolled for 30 min at 4 °C on a benchtop rolling system before being centrifuged for 20 min at 18,000 rpm at 4 °C. The α-synuclein-containing pellet was resuspended in 25 mM Tris–HCl (pH 7.7) and dialysed using a 3500 MWCO membrane in 4 L 25 mM Tris–HCl (pH 7.7). α-Synuclein was purified by ion-exchange on a Q-Sepharose™ HP HiScale™ 26/20 column (Cytiva, formerly GE Life Healthcare, United States) before size exclusion on a HiLoad™ 16/600 Superdex™ 75 pg column (Cytiva, formerly GE Life Healthcare, United States) into the appropriate experimental buffer. To determine the concentrations in solution, we used the absorbance value of the protein measured at 275 nm and an extinction coefficient of 5,600 M^−1^ The protein solutions were divided into aliquots, flash frozen in liquid N_2_ and stored at −80 °C until required for use.

#### Seed fibril preparation

Seed fibrils were produced as described previously. 500 μL samples of α-synuclein at 100–200 μM concentrations were incubated in 20 mM phosphate buffer (pH 6.5) for 48–72 h at ≈ 40 °C and stirred at 1,500 rpm with a Teflon bar on an RCT Basic Heat Plate (IKA, Staufen, Germany). Fibrils were divided into aliquots, flash frozen in liquid N_2_and stored at −80°C until required for use. For experiments at pH 6.5 (utilising μM fibril concentrations), the fibril stock was sonicated for between 0.5 and 1 min using a probe sonicator (Bandelin, Sonopuls HD 2070; Berlin, Germany), using 10% maximum power and a 50% cycle. For experiments at low pH utilising nM fibril concentrations, the fibril stock was diluted to 10 μM in water, sonicated 3 times for 5 s at 10% maximum power and 50% cycles using the probe sonicator.

#### Lipid vesicle preparation

DMPS powder was dissolved to a final concentration of 5 mM in 20 mM sodium phosphate buffer at pH 6.5 and stirred at 900 rpm at 50°C for 2–2.5 h followed by five freeze-thaw cycles in dry ice and a hot water bath at 45°C to ensure unilamellarity. To form vesicles, the solution was sonicated by a Sonopuls HD 2070 probe sonicator (Bandelin) for 3 × 5 min cycles on a 50% cycle at 10% maximum power, and centrifuged at 15,000 rpm for 30 min at 25°C to remove residue formed during sonication.

### Measurement of aggregation kinetics

Wild-type α-synuclein was incubated at the concentrations and conditions indicated and in the presence of 50 μM ThT and preformed α-synuclein fibrils at 37 °C (12, 15, 34). The change in the ThT fluorescence signal was monitored using a Fluostar Optima or Polarstar Omega fluorescence plate reader (BMG Labtech, Aylesbury, United Kingdom) in bottom reading mode under quiescent conditions. Corning 96 well plates with half-area (3,881, polystyrene, black with clear bottom) non-binding surfaces sealed with aluminium sealing tape were used for each experiment.

### Fluorescence polarisation to measure lipid fluidity

Diphenylhexatriene (DPH) was dissolved in absolute ethanol to a concentration of 2 mM and stirred at 1,500 rpm with a magnetic stir bar for 60 h at RT in darkness. DMPS vesicles were prepared as described previously to a concentration of 2 mM monomer equivalents. The vesicles (700 μM) were mixed with 3.5% volume of the 2 mM DPH solution in 20 mM Sodium Phosphate buffer (1 mM EDTA, pH 6.5) and left in darkness for 45 min. For measurement of fluorescence polarisation, samples were diluted to a final concentration of 100 μM DMPS, 0.5% volume DPH, with the molecule at the desired concentration at 1% volume DMSO. Samples were incubated for 15 min in darkness before measurement of fluorescence polarisation on a Clariostar plate reader (BMG Labtech, Aylesbury, United Kingdom) with excitation wavelength 360 nm and emission wavelength 440 nm.

### Kinetic analysis of α-synuclein aggregation

#### Fibril elongation rate

In experimental conditions under which α-synuclein fibril elongation is favoured, and where primary and secondary nucleation events are negligible, the initial rate of fibril elongation mat be approximated with a linear function (Flagmeier et al., 2016):

$${\left.\frac{dM\left(t\right)}{dt}\right|}_{t=0}=2k_{+}P\left(0\right)m\left(0\right)$$ (1)

Where *M*(*t*) is the fibril mass concentration, *P*(*0*) is the initial number concentration of fibrils, *m*(*0*) is the initial monomer concentration and k+ is the rate of fibril elongation. If *P*(*0*) and m(0) are universal for each condition tested, the relative rate of elongation for each condition may be found by normalising the value of $2k_{+}P\left(0\right)m\left(0\right)$ , that is, the slope of the initial linear points of the elongation reaction, of a given condition to that of the control condition.

#### Secondary nucleation rate and oligomeric flux

Under conditions where α-synuclein secondary nucleation is favoured, the aggregation may be described by fitting a generalised logistic function to the normalised aggregation data:

$$\frac{M\left(t\right)}{m_{tot}}=1-{\left[1+\frac{a}{c}e^{\kappa t}\right]}^{-c}$$ (2)

where *m*_*tot*_ refers to the total α-synuclein monomer concentration present within a reaction. The terms a , κ and c are fitting parameters with

$$a=\frac{\lambda^{2}}{2\kappa^{2}}$$ (3)

and

$$c=\sqrt{\frac{2}{n_{2}\left(n_{2}+1\right)}}$$ (4)

with c fixed at a value of 0.3. This corresponds to a reaction order of n2=4 , and represents secondary nucleation behaviour as observed in the case of the islet amyloid polypeptide precursor (IAPP) protein (Michaels et al., 2016). The terms λ and κ represent combinations of primary and secondary nucleation rate constants, respectively (Michaels et al., 2016).

Fitting the generalised logistic function (Eq. 2) to normalised secondary nucleation data provides an analytical approximation for the monomer concentration at time *t*, *m*(*t*), since

$$m\left(t\right)=1-M\left(t\right)$$ (5)

This, in turn, facilitates the approximation of the fibril number concentration, *P*, over time by rearranging Eq. 1 in terms of the first derivative of *P*(*0*), which corresponds to the change in fibril number over time, $\frac{dP\left(t\right)}{dt}$ . This is solved at the half-time of each reaction, where it is assumed to me maximal, to yield

$$\frac{dP\left(t\right)}{dt}={\left({\left.\frac{1}{m_{tot}}\cdot \frac{dm\left(t\right)}{dt}\right|}_{t=t_{\frac{1}{2}}}\right)}^{2}\cdot \frac{4}{k_{+}}$$ (6)

$\frac{dP\left(t\right)}{dt}$ , obtained at the half-time of the reaction, is used as an effective rate of fibril amplification and is reported for each condition relative to the control condition specified.

#### Oligomeric flux approximations

The theoretical oligomeric flux data were fitted as previously described (Staats et al., 2020). The flux towards oligomeric species over time, $\phi \left(t\right)$ , was obtained analytically by means of the following equation derived from the linear polymerisation model (Eq. 1):

$$\phi \left(t\right)=\frac{1}{r_{+}}\cdot \left[\frac{m\left(0\right)}{m\left(t\right)}\cdot \frac{d^{2}M\left(t\right)}{dt^{2}}+\frac{m\left(0\right)}{m{\left(t\right)}^{2}}{\left(\frac{dM\left(t\right)}{dt}\right)}^{2}\right]$$ (7)

where M(t) and m(t) are represented analytically in terms of the fit of the generalised logistic function (Eq. 2) to secondary nucleation data, while r_+_ denotes the effective elongation rate obtained for each compound dose as approximated by Eq. 1.

To approximate the parameters of the flux towards oligomeric species, Eq. 2 may be substituted into Eq. 7 to yield an expression for $\phi \left(t\right)$ in terms of the generalised logistic function:

$$\phi \left(t\right)=\frac{a\kappa^{2}e^{\kappa t}}{2r_{+}{\left[1+\frac{a}{c}e^{\kappa t}\right]}^{2}}$$ (8)

from which the area under the curve of the flux towards oligomeric species may be calculated by calculating the integral of Eq. 8:

$$A=\int_{0}^{\infty }\phi \left(t\right)dt=\frac{m\left(0\right)\kappa c^{2}}{r_{+}\left(a+c\right)}$$ (9)

Eq. 8 may also be used to approximate the peak time and peak height of $\phi \left(t\right)$ , since the peak time may be calculated as a solution to

$${\left.\frac{d\phi }{dt}\right|}_{t=t_{Peak}}=0\Rightarrow t_{Peak}=\frac{1}{\kappa }\ln\left(\frac{c}{a}\right)$$ (10)

which yields a description for the peak height of ϕ as

$$\phi_{Peak}=\phi \left(t_{Peak}\right)=\frac{m\left(0\right)\kappa^{2}c}{4r_{+}}$$ (11)

#### Lipid-induced aggregation analysis

Kinetic traces of lipid-induced reactions were normalised to the plateau values and the exponential initial phases of the reaction, where heterogenous primary nucleation is still dominant (Galvagnion et al., 2015), were analysed by globally fitting an approximation of one-step heterogenous primary nucleation to the data (Galvagnion et al., 2015)

$$M\left(t\right)=\frac{K_{M}k_{n}k_{+}{m\left(0\right)}^{n+1}bt^{2}}{2\left(K_{M}+m\left(0\right)\right)}$$ (12)

which describes the mass concentration of fibrils formed over time, $M\left(t\right)$ , in terms of the Michaelis constant, $K_{M}$ , which refers to the monomer concentration at which the system becomes subject to saturation effects (Buell et al., 2010), and is fixed at 125 μM, as previously determined by global kinetic analysis (Galvagnion et al., 2015). Furthermore, the equation accounts for the initial monomer concentration within the system *m*(*0*), the reaction order of the nucleation event, *n*, which is fixed at 2 (Flagmeier et al., 2016), and the concentration of α-synuclein monomer bound to the lipid vesicle surface, *b*, which is calculated as a measure of $\frac{\left[DMPS\right]}{L}$ , where *L* represents the stoichiometry of DMPS molecules on the vesicle surface interacting with one α-synuclein molecule (previously calculated by circular dichroism), and is fixed at *b* = 3.3 μM for this work (Galvagnion et al., 2015; Flagmeier et al., 2016). Fits were conducted using the online aggregation fitter, Amylofit, which is based on a basin-hopping algorithm (Meisl et al., 2016).

#### Absorbance and fluorescence emission determination

Samples were prepared to match the conditions used in the pH 5.5 aggregation kinetics described above, in the presence/absence of ThT and fibrils. The molecule was added at the desired concentration at 1% vol DMSO and absorbance and fluorescence (excitation wavelength: 440 nm) spectra were measured on a Clariostar plate reader (BMG Labtech, Aylesbury, United Kingdom), using the same plate format as was employed in the kinetic experiments.

#### Nephelometry measurements

Wild-type α-synuclein was incubated at the indicated concentration in the presence of 0.06% seed fibrils. Samples were prepared in Corning 96 well plates with half-area (3,881, polystyrene, black with clear bottom) and non-binding surfaces at a working volume of 150 µL. Plates were fitted with a transparent cover. The change in scattering due to α-synuclein aggregation was monitored using a Nephelostar Plus (BMG Labtech, Aylesbury, United Kingdom) under quiescent conditions, using a laser intensity setting of 40% and a beam focus of 1.5 mm.

### Mass spectrometry

10 μM of preformed α-synuclein fibrils were incubated with the concentrations of compound 69.2 indicated in 20 mM sodium phosphate buffer (pH 4.8) overnight under quiescent conditions at room temperature. The samples were then ultracentrifuged at 120 krpm for 30 min and the supernatant was removed for analysis using a Waters Xevo G2-S QTOF spectrometer (Waters Corporation, MA, United States).

#### Fitting of mass spectrometry dose response curves

Mass spectrometry data for fibril binding were fit with a 4-parameter sigmoidal dose response curve:

$$R=R_{\mathrm{Min}}\frac{{\left([L\right]}^{H})\left(R_{\mathrm{Max}}-R_{\mathrm{Min}}\right)}{{\left[L\right]}^{H}+K_{d_{app}}^{H}}$$ (13)

where the response, *R*, may be described in terms of the minimum and maximum response, $R_{\mathrm{Min}}$ and $R_{\mathrm{Max}}$ , respectively, as well as the ligand concentration, [*L*]. Upon fitting to the data, the Hill slope, *H*, and the apparent dissociation constant, ${K_{d}}_{app}$ are obtained.

## Data Availability

The original contributions presented in the study are included in the article/Supplementary Material, further inquiries can be directed to the corresponding author.
