# Supplementary material for: Optimization of a small molecule inhibitor of secondary nucleation in α-synuclein aggregation
Source: Front Mol Biosci. 2023 Aug 28;10:1155753. doi: 10.3389/fmolb.2023.1155753 (PMC10493395; doi:10.3389/fmolb.2023.1155753)
Supplement: Supplementary file 1 [file DataSheet1.PDF]

Supporting Information

Optimization of a small molecule inhibitor of secondary nucleation in α-synuclein aggregates

Roxine Staats, Z. Faidon Brotzakis, Sean Chia,
Robert I. Horne, Michele Vendruscolo

*Centre for Misfolding Diseases, Yusuf Hamied Department of Chemistry,
University of Cambridge, Cambridge CB2 1EW, UK*

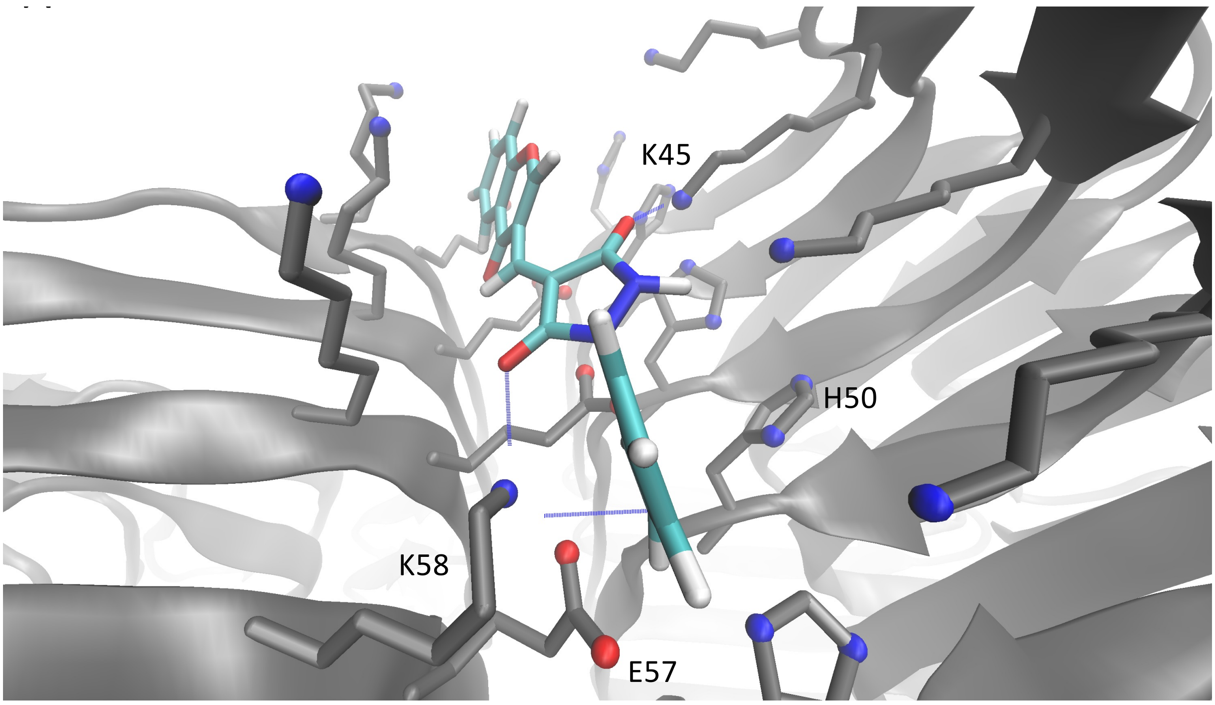

Figure S1. Docking of the parent compound 69.0 on a fibril structure of α-synuclein. The best docking pose is shown, with the respective interactions within a pocket on the α-synuclein fibril surface (PDB 6CU7) comprising residues K43, K45, H50, E57, K58.

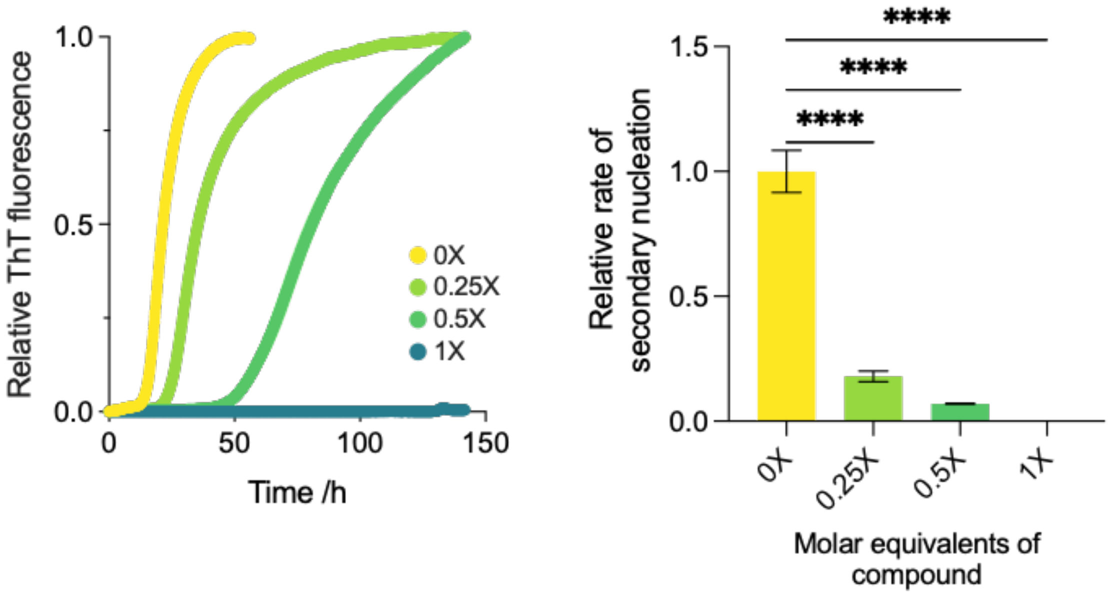

Figure S2. The parent compound 69.0 inhibits α-synuclein secondary nucleation. (A) Change in ThT fluorescence when 20 μm α-synuclein monomer was incubated at 37 °C under quiescent conditions with 0.25% seed fibrils in sodium phosphate buffer (20 mm, pH 4.8, 1% DMSO) with increasing concentrations of the parent compound 69.0 as indicated. Traces indicate the mean and error of three technical repeats. **(B)** Effective rate of α-synuclein fibril amplification, normalised relative to the DMSO control. Error bars represent the standard deviation from the mean of three technical replicates. Statistical analyses represent an ordinary one-way ANOVA result where **** represents a multiplicity-adjusted P value of ≤ 0.0001.

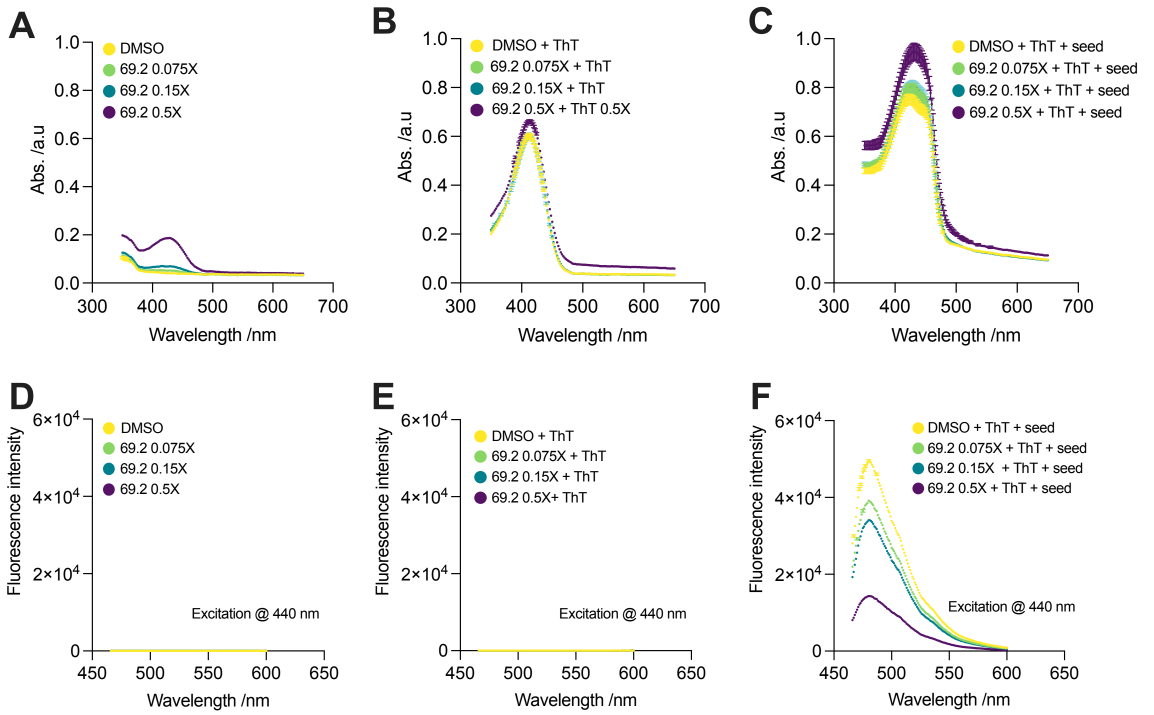

Figure S3. Absorbance and fluorescence emission analysis of compound 69.2. (A-C) Absorbance spectra of DMSO and compound 69.2 alone, with ThT and with ThT and seed fibrils. **(D-F)** Emission spectra (upon excitation at 440 nm) of DMSO and compound 69.2 alone, with ThT and with ThT and seed fibrils. All conditions in these analyses mimic those of the secondary nucleation experimental procedure.

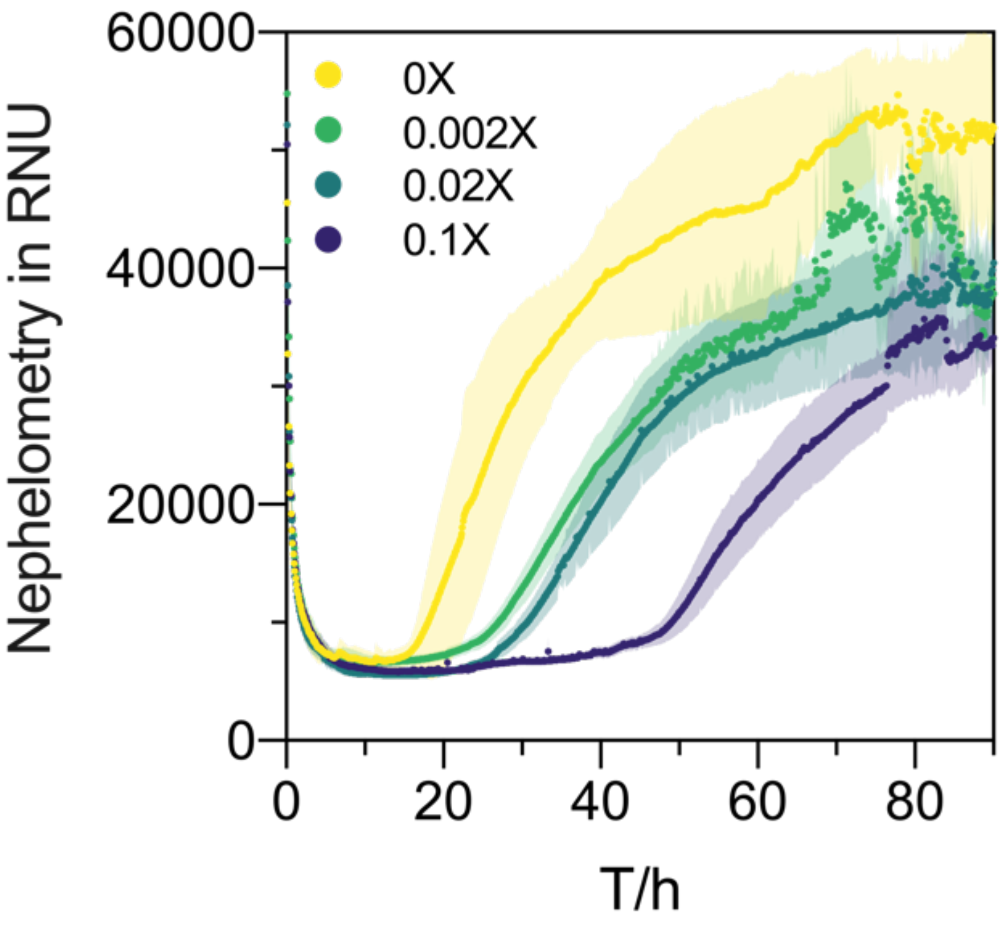

Figure S4. Label-free validation of secondary nucleation inhibition by molecule 69.2. The assay was carried out using nephelometry, which does not require the use of labels (see Methods).
